# Epithelial-Myoepithelial Carcinoma of the Palate: Report of a Case and Review of the Literatures

**DOI:** 10.30699/ijp.2020.105039.2076

**Published:** 2019-12-26

**Authors:** Nazanin Mahdavi, Maedeh Ghorbanpour

**Affiliations:** 1 *Department of Oral and Maxillofacial Pathology, School of Dentistry, Tehran University of Medical Sciences, Tehran, Iran*; 2 *Department of Oral and Maxillofacial Pathology, Faculty of Dentistry, Tehran Medical Sciences, Islamic Azad University, Tehran, Iran*

**Keywords:** Clear cell tumors, Epithelial-myoepithelial carcinoma, Head and neck, Oral mucosa, Salivary gland tumor

## Abstract

Epithelial-myoepithelial carcinoma (EMC) is considered as a rare malignant salivary gland neoplasm with good prognosis, low recurrence rate and rare metastasis. Here we present a case of epithelial-myoepithelial carcinoma in a 42-year-old female with a swelling of 3-year duration in her palate. Histopathologic evaluation of the lesion demonstrated a well-circumscribed, biphasic salivary gland tumor composed of double-layered ductal/glandular structures, composed of small luminal eosinophilic cells and abluminal larger clear myoepithelial cells, and luminal cells were positive for pan-cytokeratin, while the abluminal cells exhibited strong immunoreactivity for p63. Ki-67 proliferative index was 1% in abluminal cells. In this article, histopathologic and immunohistochemical features of EMC and its mimics are discussed and the previously reported cases of EMC in the literature are summarized.

## Introduction

Epithelial-myoepithelial carcinoma (EMC) is considered as a rare low-intermediate grade malignant salivary gland neoplasm. This entity accounts for 1% of all salivary gland tumors (1). Most cases occur in the parotid gland, however, other sites including the minor salivary glands (specially the palate), maxillary sinus, trachea, larynx, and hypopharynx are also involved (1). This tumor has a slight female predilection and the mean age of patients at diagnosis is 60 years (2).

EMC is a low-grade malignancy (3) with good prognosis, low recurrence rate and rare metastasis (4).

This paper presents an Epithelial-myoepithelial carcinoma of the palate which the diagnosis was confirmed by immunohistochemistry.

## Case Report

A 42-year-old female referred to the department of oral and maxillofacial surgery with chief complaint of swelling in her palate. The swelling was present from 3 years ago with no change in size and color of the mucosa. 

The patient was non-smoker, non-drinker, and she had no history of any systemic disease or any drug history.

On clinical examination, a sessile nodule measuring 2x2 cm was noted on left side of hard palate, area of first and second molar tooth. The overlying mucosa was intact and normal in color. The lesion was soft in consistency, and in palpation it was nontender. She stated no history of pain, paresthesia, or dysphagia. As well, there was no evidence of any lymphadenopathy on palpation. 

Incisional biopsy was performed for the lesion. Grossly, the lesion revealed a spherical elastic tissue with nodular surface projections. 

Microscopic examination of the specimen demonstrated a well-circumscribed neoplasm with salivary gland origin. The tumor had invaded the capsule by forming large sheets as well as small islands. The neoplasm was composed of islands, nests, and sheets of clear and eosinophilic cells that made scattered small duct-like structures lined by two layers of cells: the inner layer was composed of small cuboidal cells with eosinophilic cytoplasm, and the outer layer demonstrated large cells with clear cytoplasm. Tumor cells also revealed areas with oncocytic metaplasia in some parts and abundant deposition of hyalinized material in other areas (Figures 1A, B). 

A differential diagnosis including pleomorphic adenoma (PA), clear cell type of mucoepidermoid carcinoma (MEC) and epithelial myoepithelial carcinoma was suggested according to the histological findings. For the definitive diagnosis immunohistochemical (IHC) studies for ki-67, pan-cytokeratin and p63 was performed. The abluminal cells revealed strong immunoreactivity for p63 (Figure 2A), and luminal cells were positive for pan-cytokeratin (Figure 2 B). In addition, Ki-67 proliferative activity was positive in 1% of abluminal cells. 

Therefore, a diagnosis of epithelial-myoepithelial carcinoma was confirmed based on the biphasic nature of the tumor.

**Fig. 1:**
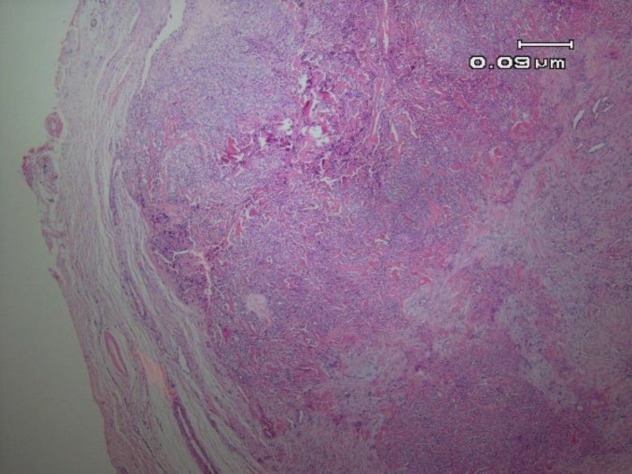
**A** **. **A well-circumscribed tumor composed of a mixture of eosinophilic and clear epithelial cells (H & E, 40X)

**Fig. 1. B:**
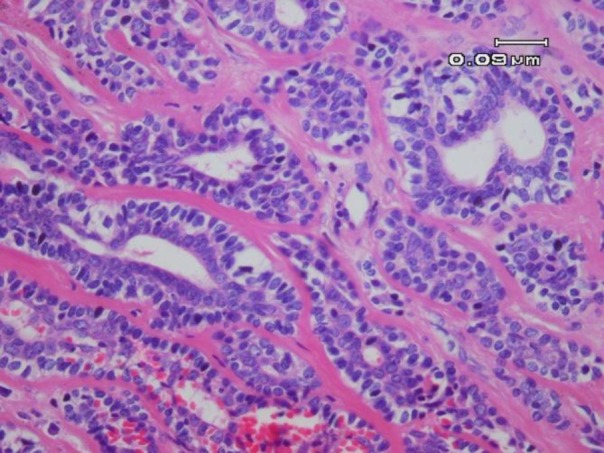
Duct-like structures lined by one layer of epithelial cells with eosinophilic cytoplasm surrounded by a layer of clear myoepithelial cells within a hyalinized stroma (H & E, 400X)

**Fig. 2. A:**
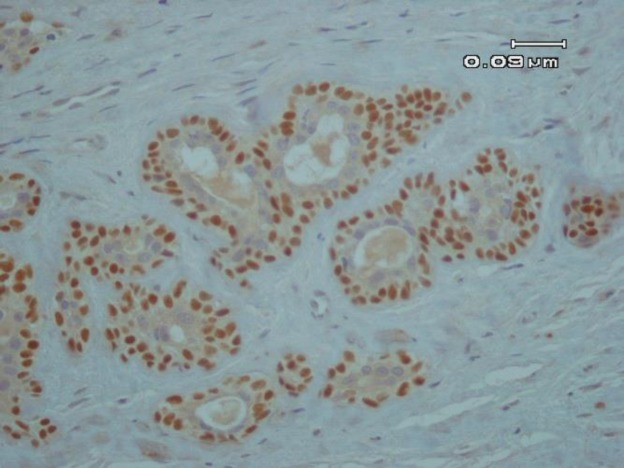
Positive nuclear immunohistochemical expression of p63 in the abluminal cells (IHC, 400X).

**Fig. 2. B:**
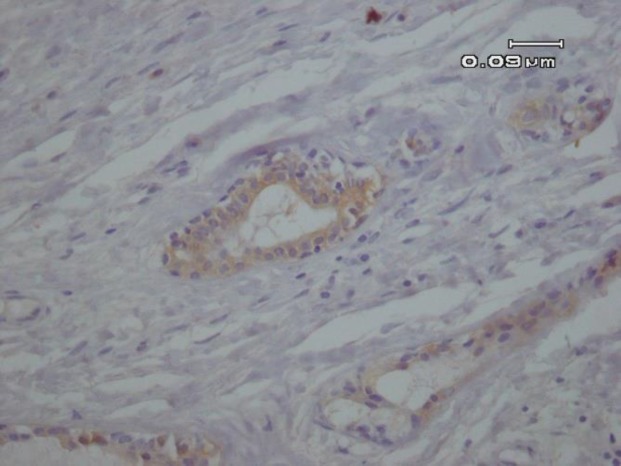
Immunohistochemical expression of CK7 reveals cytoplasmic expression in the luminal cells (IHC, 400X).

**Table 1:** Previously reported cases of epithelial-myoepithelial carcinoma arising in the palate

Immunohistochemistry		Histopathologic Features	Clinical Presentation	Sex	Age (years)	year	Reference
Positivity in Myoepithelial cells	Positivity in Ductal cells						
S-100 α-SMA	Pan keratin	Proliferation of double-layered duct-like structures with 2 distinctive cell types. The inner layer of eosinophilic epithelial cells, and outer layer of clear cells	Swelling since 20 years ago	M	72	1996	Kusama *et al *(7)
GFAP	CK	Multiple tubular or solid nests separated by a basement membrane and consisted of variable proportions of 2 cell types, cuboidal epithelial and clear myoepithelial cells	-	F	72	2000	Li *et al *(8)
S-100	CK	Round to oval cells with moderate amount of pale pink to clear cytoplasm and round to oval eccentrically placed mildly pleomorphic vesicular nuclei with small prominent nucleoli, occasional mitotic figure, some nodules showed central necrosis	Nodular swelling with surface ulceration on hard palate, no history of pain, no evidence of cervical lymphadenopathy	F	36	2008	Pai *et al *(9)
P63 calponin S100	CK AE-1/AE-3EMA CK5/6	2 cell types, an inner layer of cuboidal eosinophilic duct-like cells & an outer layer of polygonal myoepithelialcells with hyalinized stroma	A painless & asymptomatic mass with intact mucosa on hard palate	M	83	2009	Angiero *et al *(10)
p63 calponin S100 SMA	CK AE-1/AE-3 CK7 EMA CK5/6		A mass in palate & history of intermittent pain, no cervical lymphadenopathy	F	58	2009	Angiero *et al *(10)
α-SMA	CK7CK14	Double-layered duct-like structures with an inner layer of small cuboidal to oval epithelial cells with a central hyperchromatic nucleus and outer layer of polygonal with more abundant, clear, vacuolated and well-defined cytoplasm myoepithelial cells	A growing mass with regular surface & erythematousfrom 1 year ago on soft palate, difficulty in breath	F	76	2010	Muniz Alves * et al *(11)
α-SMA Calponin p63	CK7	Duct-like structures with luminal and clear abluminal cells, minimal nuclear atypia, no perineural/ angiolymphatic invasion	A mass on soft palate, speech problems,dysphagia from 2 months ago	F	66	2015	Dimitrijevic *et al *(6)
CK14 Vimentin p63 α-SMA	Pan- CK CK7 focally CK14	Eosinophilic duct-forming cells which are polygonal in shape witheosinophilic cytoplasm surrounded by clear cells, moderate nuclear atypia, no perineural/ angiolymphatic invasion	As a component of ca-ex-pa	M	42	2015	Sedassari *et al *(2)
		Same as above features except for mild nuclear atypia	As a component of ca-ex-pa	F	56	2015	Sedassari *et al *(2)
S-100 α-SMA	CKs	Biphasic structure consisting of duct-lining cuboidal cells in the inner layer and clear myoepithelial cells in the outer layer	Painless swelling of 2 months duration	M	40	2016	Pereira *et al *(12)
P63	Pan- CK	A well-circumscribed, biphasic salivary gland tumor composed of double-layered ductal/glandular structures, composed of small luminal eosinophilc cells and abluminal larger clear myoepithelial cells	A sessile nodule on left side of hard palate with no history of pain, paresthesia, or dysphagia	F	42	2019	The present case

M: male, F: female, Ca-Ex-PA: carcinoma-ex-pleomorphic adenoma, CK: cytokeratins, α-SMA: α-smooth muscle actin, EMA: epithelial membrane antigen, GFAP: glial fibrillary acidic

**Table 2:** Differential immunohistochemistry in epithelial-myoepithelial carcinoma and the mimics

Diagnosis	CK		EMA	CEA	CAM5.2	p63	Calponin	H-caldesmon	α-SMA	MSA	SMM	S100	Vimentin	GFAP	c-KIT	PLAG1	RCC/CD10
PA		+	+	+	N/A	+	+	N/A	+	+	+	+	+	+	+/-	+	-
AdCC		+	+	+	N/A	+	+	N/A	+	N/A	+	+	N/A	-	+	-	-
HCCC		+	+	N/A	+	+	-	-	-	-	-	-	+	-	N/A	-	-
MEC		-	+	+	-	+	-	N/A	-	-	-	+/-	+/-	+/-	+/-	-	-
ACC		+	+	+	+	-	-	N/A	-	N/A	-	+	+	+	+/-	-	-
MRCC		-	N/A	-	N/A	-	-	N/A	-	-	-	N/A	+	N/A	N/A	-	+
EMC		+	+	+	+	+	+	+	+	+	+	+	+	-	-	-	-

CK: cytokeratins, EMA: epithelial membrane antigen, CEA: carcinoembryonic antigen, α-SMA: α-smooth muscle actin, MSA: muscle-specific actin, SMM: smooth muscle myosin, GFAP: Glial fibrillary acidic protein, PLAG1: Pleomorphic adenoma gene 1, RCC: renal cell carcinoma, PA: pleomorphic adenoma, AdCC: adenoid cystic carcinoma, HCCC: hyalinizing clear cell carcinoma, MEC: mucoepidermoid carcinoma, ACC: acinic cell carcinoma, MRCC: metastatic renal cell carcinoma, EMC: epithelial-myoepithelial carcinoma, +: positive, -: negative, N/A: not available in the literature, +/-: rarely or focal expression (2,6,9,10,13,17,19,20,24,25)

## Discussion

 Epithelial-myoepithelial carcinoma, a rare malignant disease of salivary gland was first described by Donath *et al.* in 1972 (5). This tumor mostly develops in parotid gland. Throughout the minor salivary glands, the palate is the most common site involved by the tumor (1). The mean age of patients at diagnosis is 60 years, with a slight female predilection (2). The clinical presentation of this tumor in the parotid gland is usually an asymptomatic, slow-growing mass which may be present from several years ago, however in minor salivary glands it may exist as an ulcerated mass (6). 

Histologically, the tumor consists of varying proportion of two cell types around the duct-like structures. The inner epithelial layer cells were cuboidal to columnar with eosinophilic cytoplasm and the outer layer were myoepithelial cells with clear cytoplasm (1). Table 1 shows the clinicopathologic features and expression of different IHC biomarkers of previously reported cases of EMC in the English-language literature (2,6-12). The biphasic pattern aforementioned can be seen in some other salivary gland tumors such as benign pleomorphic adenoma, and adenoid cystic carcinoma (AdCC), but these foci are not distributed throughout the tumor (13).

The architectural pattern and relative proportion of the two cell types varies from case to case and within the same lesion (9). As well, the morphologic features of the cells in EMC overlaps with other more common benign or malignant salivary gland tumors. Therefore, this tumor may pose a diagnostic dilemma (14).

The most common tumor of salivary gland is pleomorphic adenoma (15). On microscopic examination, this tumor is composed of epithelial and myoepithelial cells, within variable stroma that may comprise of myxoid, fibrous, chondroid, mucinous, or even osseous tissue. The biphasic pattern of epithelial and myoepithelial cells in this tumor resembles EMC. In epithelial-myoepithelial carcinoma the cellular periphery of the myoepithelial portion is sharp, but in PA it gradually blends into the surrounding stroma named as "melting pattern". In addition, EMC lacks any mucinous or myxochondroid stroma (15).

AdCC is a malignant tumor composed of ductal epithelial and myoepithelial-like cells, resembling EMC in some areas. In AdCC three growth pattern (cribriform, tubular, and solid) can be observed. In addition, the neoplastic cells are basaloid and the myoepithelial cells are less frequently seen than EMC (2). 

When the clear cells predominate with a solid growth pattern, both primary and metastatic tumors with clear cells like hyalinizing clear cell carcinoma (HCCC), clear cell variants of mucoepidermoid carcinoma, acinic cell carcinoma (ACC), and, metastatic renal cell carcinoma (MRCC) are encountered in the differential diagnosis (9).

Hyalinizing clear cell carcinoma lacks a biphasic growth pattern and, unlike EMC, HCCC has prominent areas of monomorphic clear cells separated by hyalinized stroma (9). In the incisional biopsies, when sheets of a mixture of clear cells and eosinophilic epithelioid cells are the predominant feature, mucoepidermoid carcinoma is highly considered as the differential diagnosis (probably clear cell type). If EMC is misdiagnosed as MEC, then the pathologist will grade it according to MEC criteria. And Since in EMC the tumor cells are arranged in sheets and exhibit no or little cyst formation and perineural invasion is a common feature in EMC, despite being a low to intermediate grade tumor, EMC would be misdiagnosed as moderate to high grade MEC (16). As well, clear cell variant of MEC has scattered characteristic cells, such as cells with intracytoplasmic mucin, and epidermoid cells (9). In addition, identification of PAS- positive granules in ACC helps to differentiate it from EMC. MRCC lacks biphasic growth pattern and it has more nuclear pleomorphism and atypia (17). As well, the aforementioned tumors lack a myoepithelial cell component (9).

Typically, diagnosis of this tumor is based on conventional hematoxylin-eosin staining and confirmed by immunohistochemistry (4). Immunohistochemistry supports the identification of the epithelial and myoepithelial phenotypes (18). On immunohistochemical staining, the epithelial cells reveal expression of epithelial markers, including cytokeratins, epithelial membrane antigen (EMA) (4,9), carcinoembryonic antigen (CEA) (9), and CAM 5.2 (13). By contrast, the myoepithelial cells are negative for epithelial markers and demonstrate immunoexpression of myoepithelial cell markers such as p63, calponin, h-caldesmon, smooth muscle actin, muscle-specific actin, smooth muscle myosin, S100, and vimentin (18,19).

In the present study p63 was applied to highlight abluminal myoepithelial cells as it was suggested as the best myoepithelial marker in these tumor in a series of 61 cases (20). CK7 is considered as a marker of ductal luminal cells which is consistent with epithelial phenotype (21).

IHC findings confirm that the clear abluminal cells are myoepithelial cells and the luminal cells with eosinophilic cytoplasm are originated from duct cells. Some authors suggest that ductal cells are the original cells of the tumor. This is supported by the presence of multiple nodules of hyperplastic intercalated ducts in EMC of parotid (22). In the other hand, Cho *et* *al.* reported that the expression of Ki-67 in EMC is restricted to myoepithelial cells. According to this finding, they suggested a main role for myoepithelial cell in the development of EMC (23). Table 2 demonstrates the use of immunochemistry in differential diagnosis of epithelial-myoepithelial carcinoma (2, 6, 9, 10, 13, 17, 19, 20, 24, 25).

EMC is a low grade malignancy which usually demonstrates low degree of cellular atypia and mitotic activity (9). Until now, there are no definite treatment protocols for these tumors, but surgery is the treatment of choice (9). Surgical excision of the tumor with clear margins provides superior outcomes in terms of recurrence rates and survival (25). In selected cases, the surgery has been followed by adjuvant radiotherapy to avoid local recurrence (18). The efficacy of chemotherapy in the treatment is still unclear (4).

## Conclusion

EMC is a low grade malignant salivary gland tumor which demonstrates biphasic pattern in histopathologic view. This feature could be seen in different salivary gland tumors. Therefore, IHC staining for epithelial and myoepithelial cells can confirm the diagnosis and this will aid in determining the best treatment protocol.
